# Evolution of Neutralization Response in HIV-1 Subtype C-Infected Individuals Exhibiting Broad Cross-Clade Neutralization of HIV-1 Strains

**DOI:** 10.3389/fimmu.2018.00618

**Published:** 2018-03-27

**Authors:** Narayanaiah Cheedarla, Babu Hemalatha, Brahmaiah Anangi, Kannan Muthuramalingam, Murugesan Selvachithiram, Pattabiraman Sathyamurthi, Nandagopal Kailasam, Raghavan Varadarajan, Soumya Swaminathan, Srikanth Prasad Tripathy, S. Kalyanaraman Vaniambadi, D. Ramanathan Vadakkupattu, Luke Elizabeth Hanna

**Affiliations:** ^1^Department of HIV/AIDS, National Institute for Research in Tuberculosis, Chennai, India; ^2^Molecular Virology Laboratory, Molecular Biology and Genetics Unit, Jawaharlal Nehru Centre for Advanced Scientific Research, Bangalore, India; ^3^ART Center, Kilpauk Medical College and Hospital, Chennai, India; ^4^Molecular Biophysics Unit, Indian Institute of Science, Bangalore, India; ^5^Advanced Bioscience Laboratories Inc., Rockville, MD, United States

**Keywords:** human immunodeficiency virus type 1, broadly neutralizing antibodies, Indian HIV-1 subtype C infection, evolution of neutralization response, CD4-binding site, glycan-dependent neutralization

## Abstract

Strain-specific neutralizing antibodies develop in all human immunodeficiency virus type 1 (HIV-1)-infected individuals. However, only 10–30% of infected individuals produce broadly neutralizing antibodies (bNAbs). Identification and characterization of these bNAbs and understanding their evolution dynamics are critical for obtaining useful clues for the development of an effective HIV vaccine. Very recently, we published a study in which we identified 12 HIV-1 subtype C-infected individuals from India whose plasma showed potent and broad cross-clade neutralization (BCN) ability (1). In the present study, we report our findings on the evolution of host bNAb response over a period of 4 years in a subset of these individuals. Three of the five individuals (NAB033, NAB059, and NAB065) demonstrated a significant increase (*p* < 0.05) in potency. Interestingly, two of the three samples also showed a significant increase in CD4 binding site-specific antibody response, maintained stable CD4+ T cell counts (>350 cells/mm^3^) and continued to remain ART-naïve for more than 10 years after initial diagnosis, implying a strong clinical correlation with the development and evolution of broadly neutralizing antibody response against HIV-1.

## Introduction

One of the major goals of an HIV-1 vaccine is to induce broadly neutralizing antibodies (bNAbs) that can prevent HIV infection (2). The bNAbs target conserved regions in the HIV-1 envelope (Env) including conformational glycans at positions N160 and N332, the CD4-binding site (CD4BS), gp120-gp41 interface, membrane external proximal region (MPER), and fusion domain in gp41 (2–11). HIV-1 infection in the host is thought to be initiated by one or few transmitted/founder (T/F) viruses (12, 13). Shortly after infection, a strain-specific neutralizing antibody (NAb) response appears against the T/F virus (14–16). Earlier studies have reported that in adults infected with HIV-1, bNAbs usually develop after 2–4 years of infection (17). Interestingly, over a period of time, half of the chronically HIV-1-infected individuals may develop plasma neutralization breadth against 50% of HIV-1 variants (18). The autologous neutralizing response tends to be immunodominant, but when mutations occur in the variable epitopes (V1–V5) in the viral envelope due to error prone reverse transcriptase activity, it leads to virus escape from neutralization (19). Broadly cross-clade neutralizing antibodies (bNAbs) are known to develop in 10–30% of HIV-1 infected individuals. These antibodies bind to the Env trimer and neutralize a broad spectrum of globally circulating HIV-1 strains (20–23). The antibody response is thought to mature with CD4+ T cell help during the course of infection (6). Some recent studies have reported that while non-neutralizing antibodies (n-NAbs) trigger Fc-mediated ADCC, the Fc gamma receptors (FcgRs) play an important role in increasing the neutralizing activity of gp41-specific antibodies (2F5) but not gp120-specific antibodies. Therefore, modifications in the Fc domain to modulate its selective interaction with FcgRs can greatly influence the protective activity of bNAbs (24–27).

The vast majority of HIV-1 infections in India and more than 50% of HIV-1 infections globally is caused by HIV-1 subtype C [UNAIDS 2016 and NACO 2016]. Though a number of recent studies have described the identification and characterization of broadly neutralizing antibodies from HIV-1 subtype C-infected individuals (3, 7, 28–30), very few studies have examined the evolution of the broadly NAb response over time (31, 32). Recently, Patil et al. (33) studied the evolution of broadly NAb response in an elite neutralizer from India and observed an increase in the neutralization activity during follow-up. Improved neutralization activities of bNAbs during the course of evolution have also been reported from individuals infected with other clades of HIV-1 (34–36).

In a previous study, we screened a cohort of (*n* = 101) HIV-1 subtype C-infected individuals and identified 12 individuals whose plasma exhibited broad cross-clade neutralization (BCN) activity against HIV (1). In the present study, we describe the evolution of the bNAb response in a subset of these individuals in terms of breadth, potency, and neutralization specificity.

## Materials and Methods

### Ethics Statement

The study was approved by the Institutional Ethics Committee of the National Institute for Research in Tuberculosis (NIRT IEC No: 2011001), and all experiments were performed in accordance with relevant guidelines and regulations. Sample collection was done after obtaining written informed consent from the study participants.

### Sample Collection

A follow-up blood sample was obtained from 5 of the 12 individuals identified to have broad cross-clade neutralization property in our previous study (1); follow-up sample could not be obtained from the remaining 7 individuals. The follow-up time point was 4 years after the initial screening, which was within 3 years from the time of initial diagnosis of HIV infection. Blood samples were collected in tri potassium-EDTA (K_3_-EDTA) treated blood collection tubes (Ref#455036, Greiner Bio-One GmbH, Austria). Plasma was separated by centrifugation at 300 *g* and stored as 1 mL aliquots at −80°C. The plasma samples were heat inactivated at 56°C for 30 min before use.

### Estimation of CD4 + and CD8+ T Cell Counts and CD4/CD8 Ratio

CD4+ and CD8+ T-lymphocyte counts were determined on the day of sample collection, by flow cytometry as described earlier using a FACS count flow cytometer (Becton-Dickinson) (1).

### Measurement of HIV-1 Viral Load

Total HIV-1 viral load in cryopreserved plasma was estimated using the fully automated COBAS TaqMan 48 version 2.0 kit (Roche), and expressed as copies of HIV-1 RNA/ml of plasma.

### Cell Lines

293 T cells, which are human embryonic kidney cells engineered with the T-antigen from SV40 to support constant growth, were kindly gifted by Dr. Udaykumar Ranga (JNCASR, Bangalore). TZM-bl cells (Catalog Number 8129), also called JC53-BL, were obtained from the NIH AIDS Reagents Repository. This is a genetically engineered HeLa cell clone that expresses CD4, CXCR4, and CCR5 and contains Tat-responsive reporter genes for firefly luciferase and *Escherichia coli* β-galactosidase under the regulatory control of an HIV-1 Long Terminal Repeat (37, 38). Both cell lines were maintained in DMEM (Lonza, Cat# 12-604 F) containing 10% heat-inactivated fetal bovine serum (Lonza) and 50 µg gentamicin/mL (Sigma) in vented T-75 tissue culture flasks (NUNC). Cultures were incubated at 37°C in a humidified 5% CO_2_–95% air environment. Cell monolayers were split 1:10 at confluence by treatment with 0.25% trypsin, 1 mM EDTA (Sigma Cat# T4049).

### Pseudoviruses

Pseudoviruses were obtained from the NIH AIDS Reagent Repository (www.aidsreagent.org). The pseudovirus panel used in this study is provided in Table S1 in Supplementary Material. Murine leukemia virus (MuLV) env plasmid (env-pseudotyped virus containing the Env of MuLV in the same backbone vector as all HIV-1 env-pseudotyped viruses, MuLV/pSG3Δenv) was kindly gifted by Dr. V. Raghavan (IISc, Bangalore). The MuLV Env-pseudotyped virus was used as negative control in the virus neutralization assays.

### Pseudovirus Production and Titration

HEK293T cells were transiently transfected with two plasmids, one carrying the HIV-1 envelope (pEnv), and the other carrying the entire HIV-1 genome except the envelope (pSGΔenv), using the standard calcium phosphate protocol (39). Briefly, exponentially dividing 293 T cells were seeded at a concentration of 0.7 × 10^6^ cells in a T-25 tissue culture flask and incubated at 37°C in a humidified 5% CO_2_–95% air environment overnight. On the following day, old medium was replaced with fresh medium, and cells were co-transfected with the rev/env expression plasmid (5 µg) and env-deficient HIV-1 backbone vector (pSG3ΔEnv) (10 µg) using the calcium phosphate method, and incubated for 5 h at 37°C in a humidified 5% CO_2_–95% air environment. After 5 h, the entire medium was removed from the flask and fresh DMEM was added. Pseudovirus-containing culture supernatant was harvested 2 days later, filtered using a 0.45 µm pore size filter, and stored as 1 mL stocks with 20% FBS at −80°C.

The 50% tissue culture infectious dose (TCID_50_) was determined in TZM-bl cells as described by Montefiori (40). After 2 days of infection, the culture medium was removed from the wells. 100 µL of Britelite Plus reagent (Perkin Elmer) was added to each well, and the plate was incubated at room temperature (RT) for 2 min to allow complete cell lysis. The contents of the wells were mixed by pipettor action (at least two strokes) and 100 µL of cell suspension was transferred to corresponding wells of a 96-well black (opaque) plate. The plate was read immediately in a luminometer and TCID_50_ was calculated using “TCID” macro (https://www.hiv.lanl.gov/content/nab-reference-strains/htmL/home.htm).

### Neutralization Assay

The neutralization potential of the plasma samples was evaluated using the standard neutralization assay with 200 TCID_50_ pseudoviruses as described earlier (1). In order to determine the neutralization potency (ID_50_ value), serial dilutions of the plasma samples ranging from 1:20 to 1:43,740 were used. Anti-glycan neutralization activity was evaluated using Du156WT, Du156N160K, Du156N332A, JR-FLWT, and JR-FL E168K pseudoviruses. Further, polyclonal IgG was eluted from the plasma samples using tosylactivated MyOne Dynabeads coupled with 93IN101-gp145 trimer, and neutralization (IC_50_) and cell-surface binding (EC_50_) ability of the samples were determined against a tier-2 pseudovirus, JR-FL WT, and its mutant, JR-FL E168K.

### Coupling, Adsorption, and Elution of pIndie gp145-Specific Antibodies From BCN Samples

Paramagnetic polystyrene, tosylactivated MyOne Dynabeads (Life Technologies) were coupled with recombinant gp145 protein of Indie-C1 which is an Indian HIV-1 reference strain. The gp145 of Indie-C1 is codon optimized and well-studied (41). Briefly, 0.5 mg of protein was coupled with 2.50 mg of tosylactivated magnetic, i.e., MyOne Dynabeads. Coupling was done at room temperature in a total volume of 1.25 ml in coupling buffer (0.1 M sodium borate buffer [pH 9.5] containing 1 M ammonium sulfate) with gentle rocking for 48 h. The Dynabeads and bound protein were separated from the coupling buffer using a magnet and resuspended in 5 ml of blocking buffer (PBS [pH 7.4] containing 0.5% [wt/vol] bovine serum albumin [BSA] and 0.05% Tween 20) for an additional 48 h. The blocking buffer was removed by aspiration, and the protein-coupled Dynabeads were washed two times with 5 ml of wash buffer (PBS [pH 7.4] containing 0.1% BSA and 0.05% Tween 20). The beads were then resuspended in 0.5 ml of storage buffer (PBS [pH 7.4] supplemented with 0.1% BSA, 0.05% Tween 20, and 0.02% sodium azide + protease inhibitors) and stored at 4°C. On the following day, the protein-coupled beads were washed three times with DMEM containing 10% fetal bovine serum, and incubated in fresh DMEM containing 10% FBS at RT for 30 min to block nonspecific binding to the beads. All the follow-up samples were diluted 1:5 in DMEM containing 10% FBS, and 1 ml of the diluted plasma was incubated with 0.5 ml of beads at RT for 1 h. This step was repeated three times for the better removal of antibodies. Beads coupled with gp145-specific antibodies were stored in PBS containing 0.2% BSA and 0.02% sodium azide at 4°C. The protein-coupled beads were washed three times with PBS containing 500 mM NaCl and once with PBS, and the antibodies were eluted by decreasing the pH in a stepwise manner. First, the beads were mixed with 100 mM glycine-HCl elution buffer (pH 2.7) for 30 s. The acid-eluted solution containing IgG was quickly removed and placed in a separate tube containing 0.5 ml of 1 M Tris (pH 9.0) buffer so as to attain a pH of 7.0 to 7.4. This process was repeated three times. The eluted IgG was diluted in DMEM and concentrated over a 30-kDa Centricon plus filter (Millipore Corp.). Subsequently, the same procedure was performed on the beads at an elution pH of 2.2 to recover any IgG resistant to elution at pH 2.7. The IgG fractions recovered by both acid elution steps were combined and the concentration of the combined IgG fractions was measured using nanodrop technology (Thermofisher). The eluted IgG was characterized using cell-surface binding assay and virus neutralization assay.

### Cell-Surface Binding Assay

Human immunodeficiency virus type 1 envelope glycoprotein (Env) expression on the cell surface was achieved as previously described (42). Briefly, 293 T cells were transiently co-transfected with codon-optimized JR-FL wild-type or JR-FL E168K mutant Env-encoding pSVIII plasmid with a pctat plasmid at 3:1 ratio using the calcium phosphate method (1). Cells were harvested 48 h post-transfection, washed thrice with FACS buffer (PBS with 2% FBS), stained with monoclonal neutralizing antibodies or polyclonal IgG eluted from BCN samples, and incubated for 1 h at 37°C at concentrations ranging from 10 µg/ml to 0.0001 µg/ml. After washing, the cells were stained with a PE-conjugated mouse anti-human IgG secondary antibody (1:100 dilutions, BD Bioscience) for 30 min. Cells were again washed and resuspended in 0.5% paraformaldehyde (in PBS). Cell-surface binding of HIV-1 Env-specific antibodies was analyzed by flow cytometry (FACS Verse/Canto analyzer II, BD Bioscience and FlowJo software version 10.0.6, Tree Star Inc.), and binding curves were generated by plotting the mean fluorescence intensity of antigen binding as a function of antibody concentration. Known monoclonal neutralizing antibodies (MAbs) VRC01, PG9, 2G12, and 4E10 that bind specifically to the CD4BS, N160-glycan, N332-glycan, and MPER regions of the HIV-1 envelope, respectively, were used as positive controls.

### Enzyme Linked Immunosorbent Assays

#### Linear PepScan ELISA

Linear gp160 PepScan ELISA was performed as per the procedure described previously (1). Briefly, fifteen amino acid long linear peptides with 11 amino acids overlap, spanning the entire length of gp160 of the Indian subtype C virus, 93IN101 (Table S2 in Supplementary Material), were synthesized commercially (Infinity Biotech and Resource Inc., PA, USA). The peptides were adsorbed onto 96-well ELISA immunomaxisorp plates (Thermo Fisher) at a concentration of 5 µg/mL in 100 mM NaHCO_3_, pH 9.6, by overnight incubation at 4°C. The plates were washed four times with PBST (1XPBS containing 0.05% Tween20) using a plate washer (ELx50-BioTek) and blocked for 2 h at RT in PBS containing 1% BSA and 0.05% Tween 20. The plates were again washed four times, and heat-inactivated plasma samples diluted 1:50 with the diluent (ABL Inc., MD, USA) were added and incubated at 37°C for 1 h. Following another four washes, goat anti-human IgG horse radish peroxidase conjugated secondary antibody (ThermoFisher) at 1:120 K dilution was added and incubated for 45 min at 37°C. Plates were washed four times with wash buffer and developed using the One-STEP TMB (Thermo Fisher) substrate. After 30 min, the reaction was stopped with 1 N H_2_SO_4_, and the plates were read using a microplate reader (ELx808-BioTek). This experiment was performed two times for each sample and mean values were determined to represent the binding reactivity. Healthy human plasma pool (HHP) was used as the negative plasma control.

### ELISA With Conformational Proteins

ELISA was performed with 93IN101 gp145 recombinant [cytoplasmic tail (CT) of 93IN101 gp160 completely deleted for the expression of gp145 protein] trimer and dimer proteins that express intact conformational epitopes targeted by the major classes of bNAbs, namely, CD4BS, N160-V1/V2 glycan, N332- V3 glycan and MPER (41), as well as HXB2 gp120 monomer and NL4-3 gp41 monomer proteins (kindly gifted by Dr. V. S. Kalyanaraman, ABL Inc). BCN samples were tested at a dilution of 1:200 following the protocol described earlier (1). HHP was used as the negative control.

### ELISA With RSC3 and CD4 Outer Domain (CD4OD) Proteins

ELISA was performed with recombinant re-stabilized core 3 WT (RSC3) protein that is employed widely for the identification of CD4BS antibodies, a double mutant of the RSC3 protein which contains a deletion at position 371 and a substitution at position 363 (Proline substituted with Asparagine), resulting in addition of an N-linked glycan on the β15 strand near the CD4-binding loop (RSC3Δ371I/P363N), and CD4OD protein which comprises of the outer domain of the CD4 molecule that is based on the HIV-1 HXB2 sequence but with better exposure of the highly conserved CD4BS leading to better identification of CD4BS-specific antibodies that could not be identified using RSC3 protein (43–45). BCN samples were tested at a dilution of 1:200 as described previously (1). HHP was used as the negative control.

### Healthy Human Plasma Pool (HHP)

Blood samples were collected from 23 healthy HIV negative individuals in tri potassium-EDTA (K_3_-EDTA) treated blood collection tubes (Ref#455036, Greiner Bio-One GmbH, Austria). Plasma was separated by centrifugation at 300 *g* for 10 min. Plasma were pooled and stored as 1 mL aliquots at −80°C until use. This plasma was used as the negative control for all our experiments. The plasma was heat inactivated at 56°C for 30 min before use.

### Statistical Analysis

All statistical analyses were performed using Graphpad prism 5.0. Experiments were performed two or more times and values obtained from three replicate samples were averaged in each experiment. Statistical significance was tested using *t*-test. Differences were considered significant at *p* < 0.05.

## Results

### Clinical Profile of Study Subjects

Follow-up samples were obtained from five broad cross-clade neutralizers (four males and one female, aged between 35 and 45 years). Four of these individuals had CD4+ T cell counts >350 cells/mm^3^ (median 489 cells/mm^3^) at the time of follow-up. Only one had a lower CD4+ T cell count of 204 cells/mm^3^. Two of the five individuals (NAB033 and NAB059) continued to be ART-naïve till the time of the present study, while the remaining three were started on antiretroviral therapy during the intervening period. Demographic details and clinical profile of the study subjects are provided in Table 1.

**Table 1:** Clinical and demographic details of the BCN samples at the time of follow-up sample collection.

S. no	Age at			Sex	Source of infection	CD4+ T-cell count/mm^3^, month wise																						1st VL	2nd VL
						
Diagnosis	First sampling (2011)	Second sampling (2015)	0	6	12	18	24	30	36	42	48	54	60	66	72	78	84	90	96	102	108	120	126	132		
NAB001	32	37	41	F	Hetero sexual	422	417	427	359	416	447	502	471	487	431	512	374	291^#^	470	419	NA	204*						61,600	1,240,000

NAB033	28	33	37	M	Hetero sexual	814	852	793	804	737	689	NA	713	584	533	481	521	570	609	703	598	569	542	617	NA	NA	525*	97,600	609,000

NAB059	37	41	45	M	Hetero sexual	574	595	449	427	434	398	366	419	507	634	663	611	677	649	599	503	422*						380,000	443

NAB063	28	30	34	M	Hetero sexual	293	357	357	417*	369	294^#^	436	580	576	516	613	623	518*										3,520	20

NAB065	36	37	41	M	Hetero sexual	194	207	240^#^	613	479	567	544	762	NA	449	460*												83,400	<20

^#^ Indicates the time at which ART was started, * indicates the time of sampling, VL indicates viral load of the sample (viral RNA copies/mL), NA indicates CD4 cell count not available. Gray color represents individuals who are ART naïve.

### Increase in Neutralization Potency Against Tier-3 Pseudoviruses

**Figure 1:**
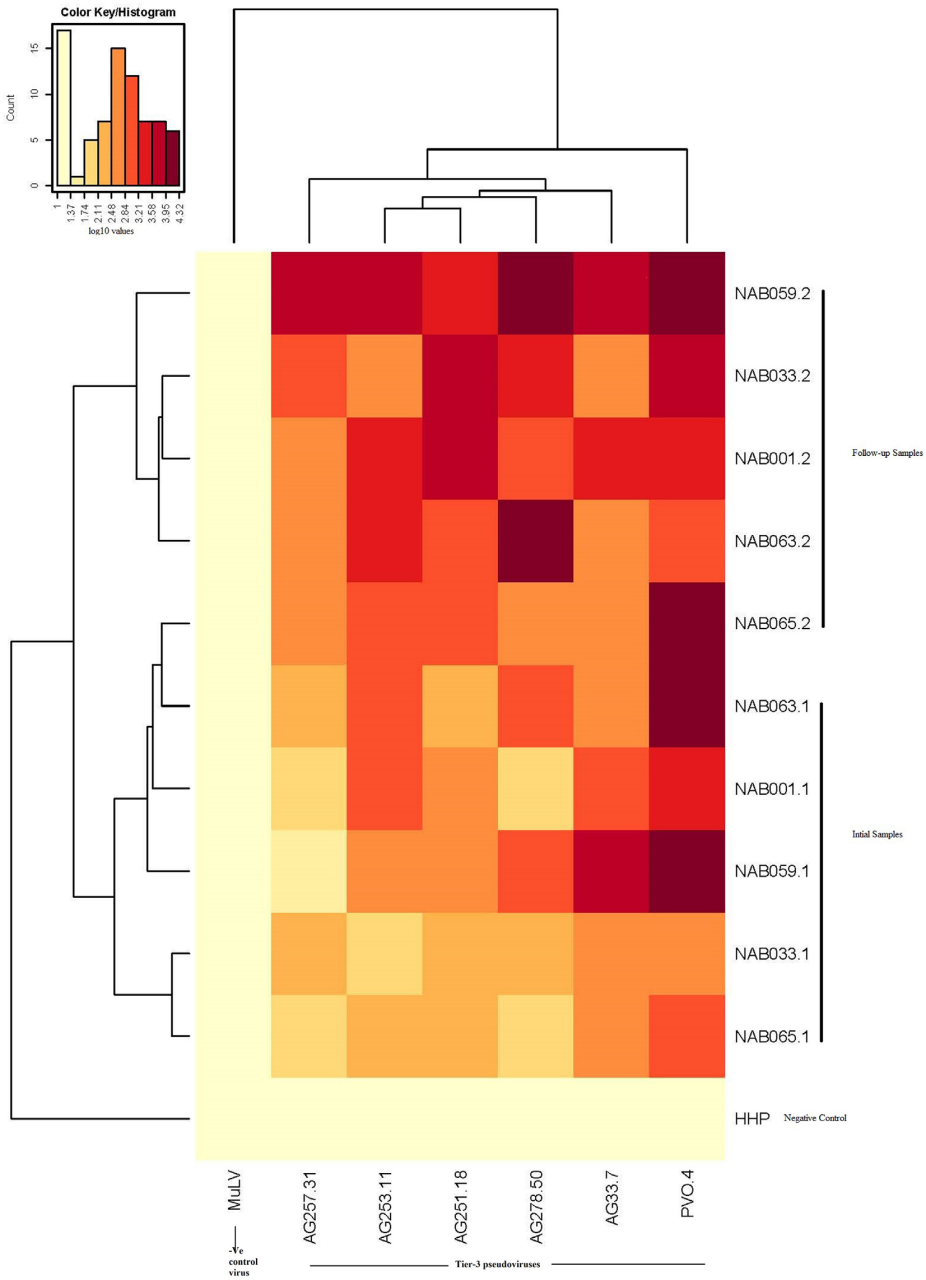
Heat map of the results of the neutralization assay of the follow-up samples tested against tier-3 pseudoviruses, and hierarchical clustering of plasma and viruses based on neutralization titers.

**Figure 2:**
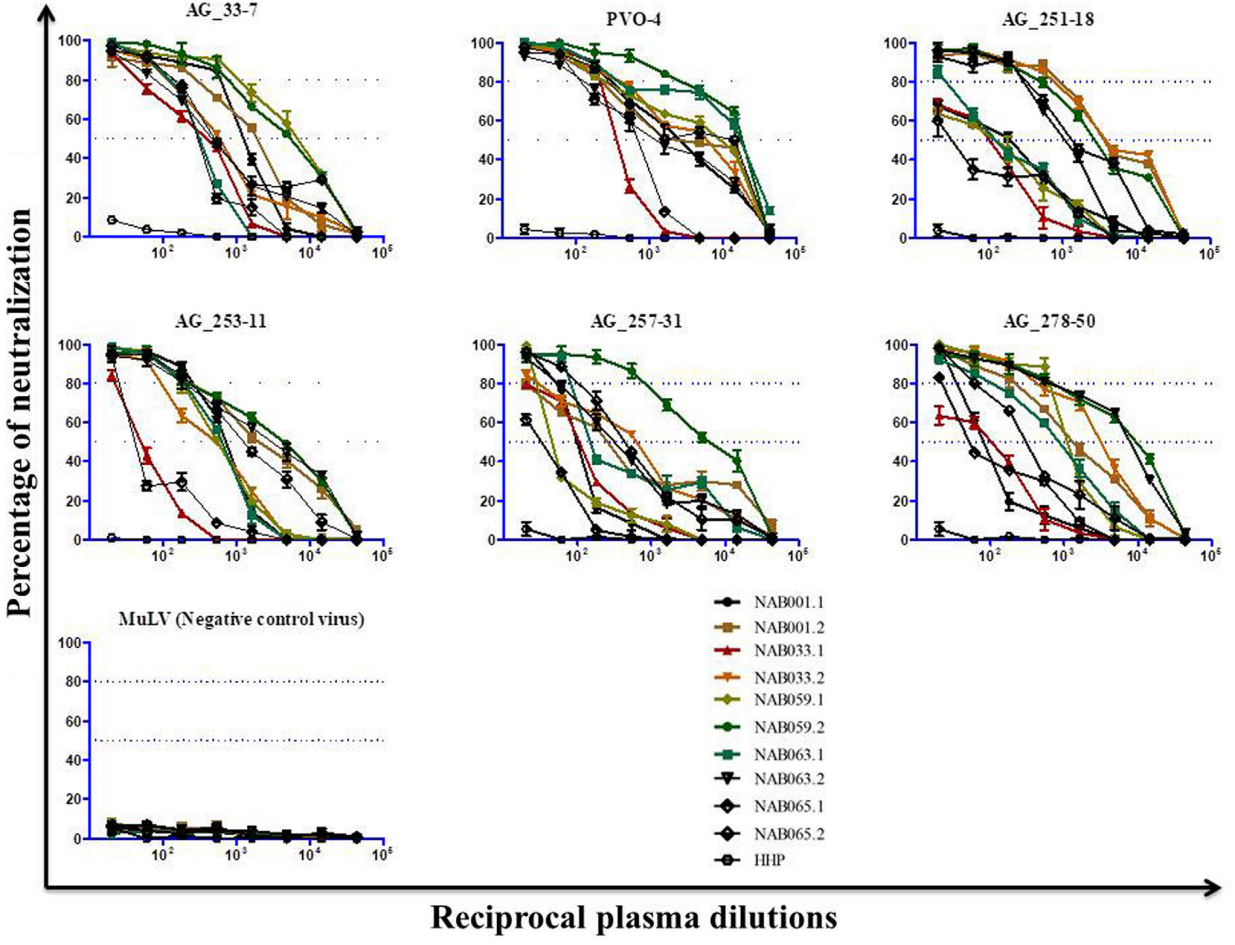
Neutralization activity of the follow-up plasma samples (*n* = 5) against tier-3 pseudoviruses. Neutralization titration (dilutions 1:20 to 1:43,740) analysis was performed against 6 tier-3 pseudoviruses in TZM-bl cells.

Since the plasma samples of the study subjects were tested previously for neutralization against tier-1, tier-2, and tier-3 pseudoviruses (1), the follow-up samples were directly tested with a panel of 6 tier-3 pseudoviruses at varying dilutions. All five samples exhibited strong neutralization potency against the tested viruses. A heat map of the log neutralization titer of the 5 samples is shown in Figure 1; the dendrogram at the top of the figure shows three clusters of two, three and one viruses, with the one on the right (PVO-4) being most sensitive to neutralization, those in the middle (251-18, 253-11, and 278-50) being moderately sensitive to neutralization and those on the left (257-31 and 33-7) being less sensitive to neutralization with the tested samples (Table 2). Results of the neutralization titer analysis of the plasma samples are represented in graphical format in Figure 2. The 50% inhibitory dilution (ID_50_) values were calculated using a dose-response curve fit with non-linear function in Graph Pad Prism, v. 5.0. The Geometric mean titer (GMT) was calculated for each sample against all the tier-3 pseudoviruses (Figure 2; Table 2). Four of the 5 samples (NAB001, NAB033, NAB063, and NAB065) neutralized all the tier-3 pseudoviruses with a GMT of 1,678; 1,803; 1,565; and 1,159, respectively. The fifth sample (NAB059) showed an extraordinarily high neutralization potency with a GMT of 7190, prompting us to categorize this individual as an elite neutralizer. Further, we compared first time point neutralization potency (1) with that seen during follow-up, and found that the neutralization potency of 3 of the 5 follow-up samples had increased significantly over time (Table 2; Figure S1 in Supplementary Material). Interestingly, two of these individuals (NAB033 and NAB059) continued to maintain stable CD4 counts and remained ART-naïve.

**Table 2:** Neutralization titration (ID_50_ and ID_80_) analysis of the second-time point plasma samples.

Sample ID	HIV-1 Tier-3 pseudoviruses												GMT		Control virus MuLV*	Fold change^#^ (follow-up GMT/initial GMT)	*p* Value
						
	subtype B		subtype AG														
							
	PVO-4		33-7		251-18		253-11		257-31		278-50						
										
	ID_50_	ID_80_	ID_50_	ID_80_	ID_50_	ID_80_	ID_50_	ID_80_	ID_50_	ID_80_	ID_50_	ID_80_	ID_50_	ID_80_			
NAB001.1	2,731	317	1,430	802	491	<20	876	447	115	46	93	30	512	173	<20	**3.27**	NS
NAB001.2	1944	252	2,228	525	4330	1,217	2,346	210	375	20	1,356	191	1,679	225	<20		
NAB033.1	417	195	506	51	220	<20	64	24	149	<20	275	<20	223	62	<20	**8.08**	
NAB033.2	6,943	224	670	146	4237	855	572	120	962	41	3,170	363	1,803	192	<20		0.0022
NAB059.1	11,016	201	8,225	981	428	<20	600	207	49	25	1,271	786	1,064	240	<20	**6.75**	0.0483
NAB059.2	20,894	3,350	5,412	608	3390	533	4,658	184	6,906	917	11,208	515	7,190	675	<20		
NAB063.1	14,915	397	365	107	175	21	718	192	165	75	1,174	104	714	105	<20	2.19	NS
NAB063.2	1,575	124	528	64	1,294	455	3,614	181	420	50	9,016	713	1,566	169	<20		
NAB065.1	855	143	361	167	296	<20	136	43	75	<20	100	29	213	74	<20	**5.44**	
NAB065.2	14,881	237	515	174	1,274	196	1,407	191	491	123	360	60	1,159	150	<20		0.0260
HHP. 1	<20	<20	<20	<20	<20	<20	<20	<20	<20	<20	<20	<20	NA	NA	<20	NA	NA
HHP. 2	<20	<20	<20	<20	<20	<20	<20	<20	<20	<20	<20	<20	NA	NA	<20		

Plasma ID_50_ and ID_80_ values are color coded as follows: no color, <100, yellow squares 100 ≤ 499, green squares 500–999, orange squares 1,000–1,999, and red squares, ≥2,000. ID_80_ values are drawn using slope factor (hillslope) values in EC anything from EC_50_ from prism. This experiment was done in duplicate on two independent occasions. NA indicates not applicable. *Excluded for GMT calculations. *MuLV, murine leukemia virus control, HHP.1 and HHP.2 – healthy human plasma pools collected at first and second time points respectively. ^#^Fold change >threefold formatted as bold. *p* values were calculated for first time point and follow-up samples ID_50_ against all tested tier-3 pseudoviruses using *t*-test.

### Increase in the Breadth of Reactivity to Env Peptides

**Figure 3:**
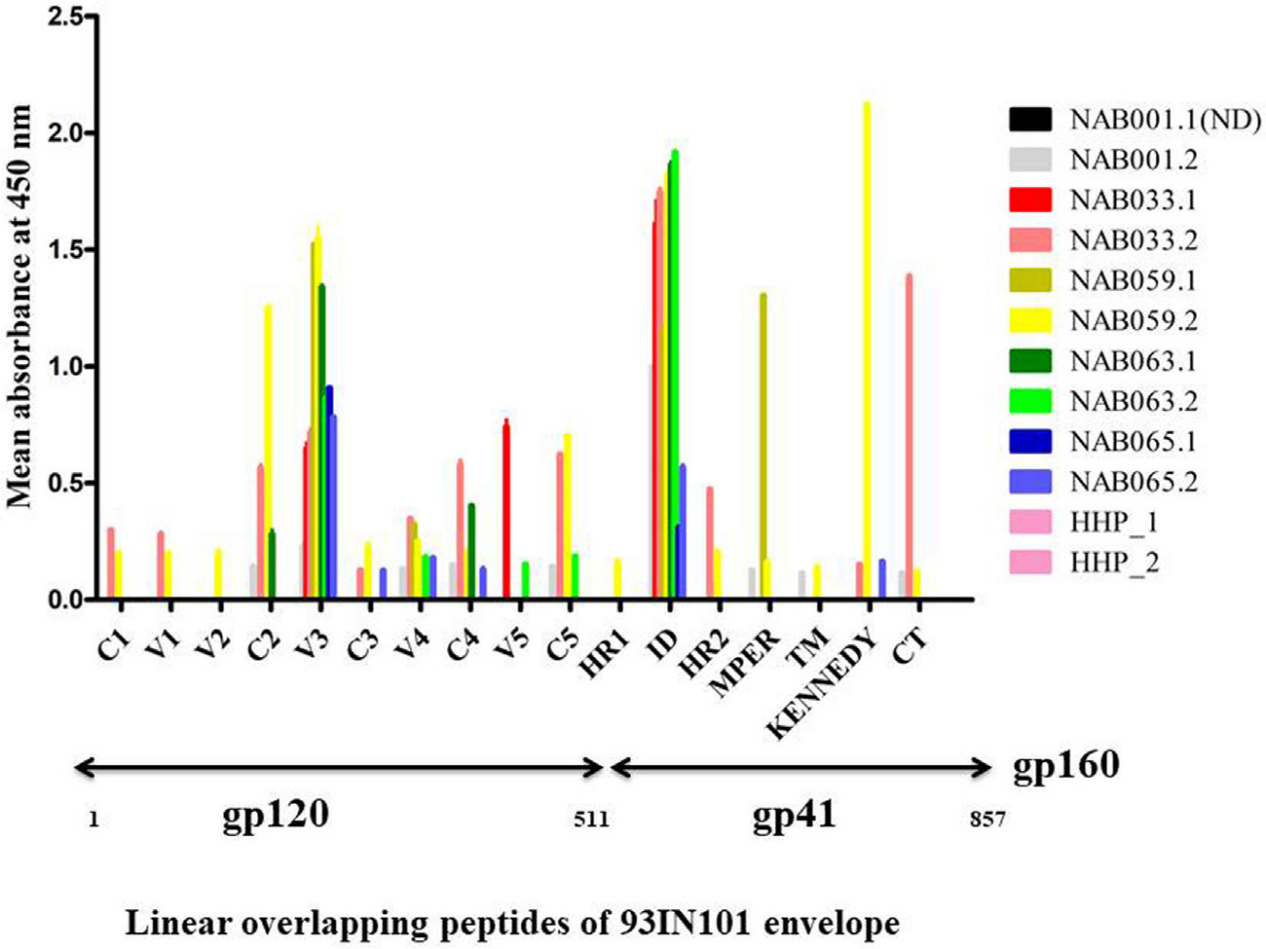
Antibody binding to linear overlapping peptides of 93IN101 Env gp160. ELISA was performed with a total of 192 peptides, 15 amino acids long with 11 amino acids overlap, covering the entire 93IN101 envelope (gp160) protein. Plasma samples from BCN samples (*n* = 5) were tested at a dilution of 1:50 for determining binding reactivity. Healthy Human Plasma pool (HHP) was used as the negative control. This experiment was performed in duplicate on two independent occasions. Mean absorbance of HHP was 0.054; twofold of HHP absorbance (0.162) was considered as positive reactivity. Normalized absorbance values are used for the graphical representation. ND indicates not done.

PepScan ELISA was performed with linear overlapping HIV-1 gp160 peptides to identify if newer antibody responses were generated in any of the BCN samples. Samples were considered reactive if the sample absorbance was two-fold more than that of the negative control (HHP) (Figure 3; Table S3 in Supplementary Material). Interestingly, samples NAB033 and NAB059, showed newer antibody responses with binding reactivity spread across the regions C2, V4, C4, C5, HR2, and CT of the envelope protein. We also found that samples NAB063 and NAB065 acquired additional binding antibody response to V4, V5, C5 and C3, C4, V4 regions of HIV-1 Env protein. As in the earlier time point, all the second time point samples also bound strongly to the V3 loop (peptide nos. 68 to 70) and Immuno Dominant region of gp41 (peptide nos.140 to 143) (Figure 3). Sample NAB059 showed strong binding reactivity to the C2 region which comprises a part of the CD4BS, and also to the HR1 and HR2 regions. NAB033 showed binding reactivity to the HR2 region. The HR1 and HR2 regions are known to play a major role in virus-host cell fusion.

### Enhanced Reactivity to Conformational Env Proteins

**Figure 4:**
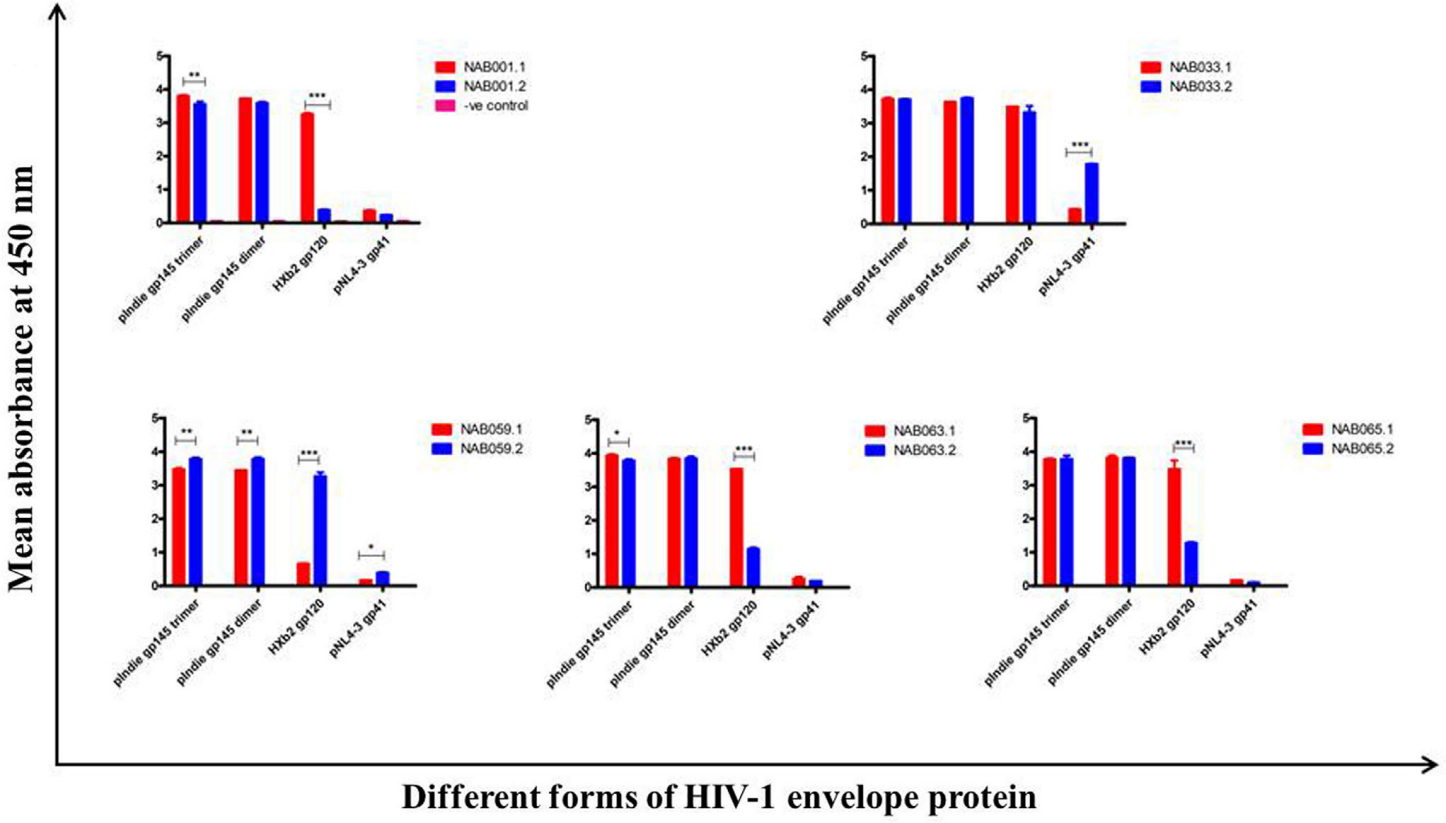
ELISA with recombinant HIV-1 envelope proteins. ELISA was performed with 93IN101 gp145 trimer, dimer, HXB2 gp120 monomer, and NL4-3 gp41 monomer proteins. All the BCN plasma samples were tested at 1:200 dilution, and HHP was used as the negative control. Each experiment was performed in triplicate on two independent occasions. * indicates *p* < 0.05, ** indicates *p* < 0.01, *** indicates *p* < 0.001.

The plasma samples were tested in an ELISA with different forms of the HIV-1 envelope protein, *viz*. 93IN101 gp145 trimer and dimer, HXB2 gp120 monomer and NL4-3 gp41 monomer, and the results were compared with that obtained at the first time point (Figure 4; Table 3). Interestingly, the second time point sample of NAB059 showed a significant increase in the binding reactivity to all four conformational proteins as compared to the previous time point (*p* < 0.05). On the other hand, NAB033 showed a significant increase in binding to the NL4-3 gp41 monomer (*p* < 0.001) but not to the other forms of the envelope protein. There was no significant increase in the binding reactivity to the conformational envelope proteins over time in the remaining samples.

**Table 3:** ELISA with different forms of HIV-1 envelope protein.

Name of the protein	BCN plasma samples									
	
	NAB001.1	NAB001.2	NAB033.1	NAB033.2	NAB059.1	NAB059.2	NAB063.1	NAB063.2	NAB065.1	NAB065.2
93IN101 gpp145 trimer	**		Ns	Ns		**	*		Ns	Ns
93IN101 gpp145 dimer	Ns	Ns	Ns	Ns		**	Ns	Ns	Ns	Ns
HXB2 gp120 monomer	***		Ns	Ns		***	***		***	
NL4-3 gp41 monomer	Ns	Ns		***		*	Ns	Ns	Ns	Ns

* indicates p < 0.05, ** indicates p < 0.01, *** indicates p < 0.001, and Ns, indicates no significant difference.

### Increase in CD4BS-Specific Antibody Response Over Time

**Figure 5:**
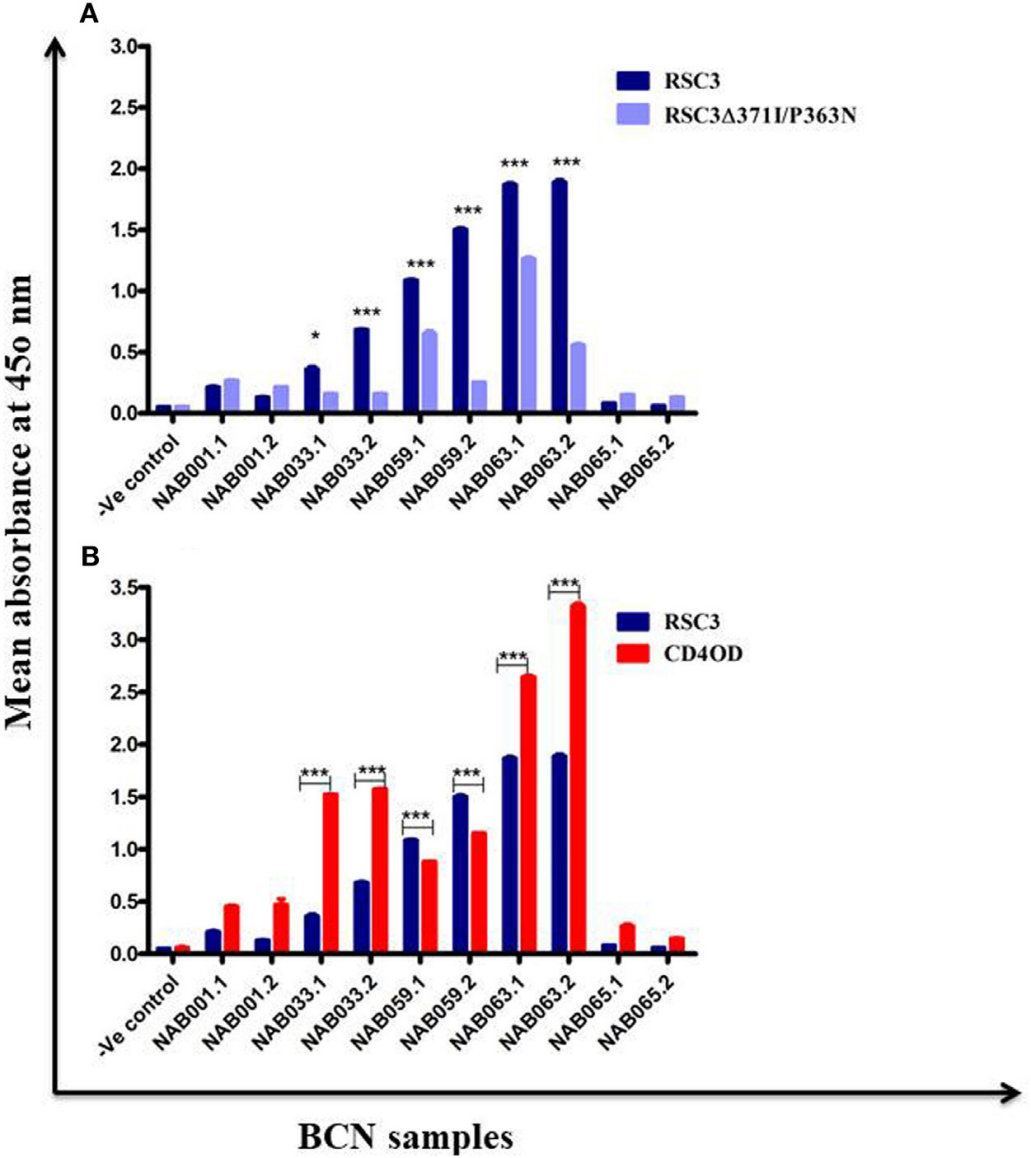
ELISA with recombinant RSC3 WT, RSC3 mutant and CD4 outer domain (CD4OD) proteins. **(A)** ELISA was performed with recombinant wild type RSC3 and double mutant RSC3Δ371I/P363N proteins. **(B)** ELISA was performed with HIV-1 subtype B-CD4OD protein and RSC3 recombinant protein. Each experiment was performed in triplicate on two different occasions. Difference was considered significant if *p* < 0.05.

The samples were tested in an ELISA with the wild-type RSC3 and double mutant RSC3Δ371I/P363N CD4BS recombinant proteins, at a dilution of 1:200 to investigate changes in CD4BS specific antibody responses in the BCN samples. Three of the five samples which showed CD4BS specificity at the initial time point again showed CD4BS-specific antibody response as revealed by greater binding response to the RSC3 wild-type protein than to the RSC3 mutant (RSC3Δ371I/P363N) protein (Table 4). When these results were compared with the corresponding results of the first time point samples, it was found that the level of RSC3 binding antibodies had significantly increased with time in two of the three samples (NAB033 and NAB059) (*p* < 0.001) (Figure 5A).

**Table 4:** ELISA with RSC3 and RSC3Δ371I/P363N proteins.

BCN sample	OD at 450 nm		*p* Values
		
	RSC3	RSC3D371I/P363N	
Neg control	0.059	0.0595	Ns
NAB001.2	0.132	0.2165	Ns
NAB033.2	0.6845	0.161	0.001
NAB059.2	1.504	0.2545	0.001
NAB063.2	1.889	0.558	0.001
NAB065.2	0.0625	0.1325	Ns

*Ns indicates no significance, samples NAB001.2 and NAB065.2 did not show binding may be changes in the sequences of RSC3 and its double mutant*.

The recombinant RSC3 protein is designed based on a HIV-1 subtype B strain (8) and has been widely used for the identification and isolation of several well-characterized CD4BS neutralizing antibodies like VRC01, VRC02, VRC03, etc. However, there is sufficient evidence to show that the CD4BS of Indian subtype C HIV-1 Env varies from that of subtype B due to differences at the molecular level (46, 47). Since all our samples were obtained from HIV-1 subtype C-infected individuals, we decided to use an additional HIV-1 envelope protein for identification of CD4BS antibodies. Saha et al. (43) designed the CD4OD protein (CD4OD) for the identification of CD4BS antibodies from HIV-1 subtype C-infected individuals. We performed ELISA with the CD4OD protein and found that four of the five BCN samples showed significantly higher (*p* < 0.01) binding reactivity to the CD4OD protein than to RSC3 (Figure 5B). In contrast, one sample (NAB059) showed significantly higher (*p* < 0.001) binding to the RSC3 protein than to the CD4OD protein.

### Increase in PG9/PG16 Like NAb Response Over Time

**Figure 6:**
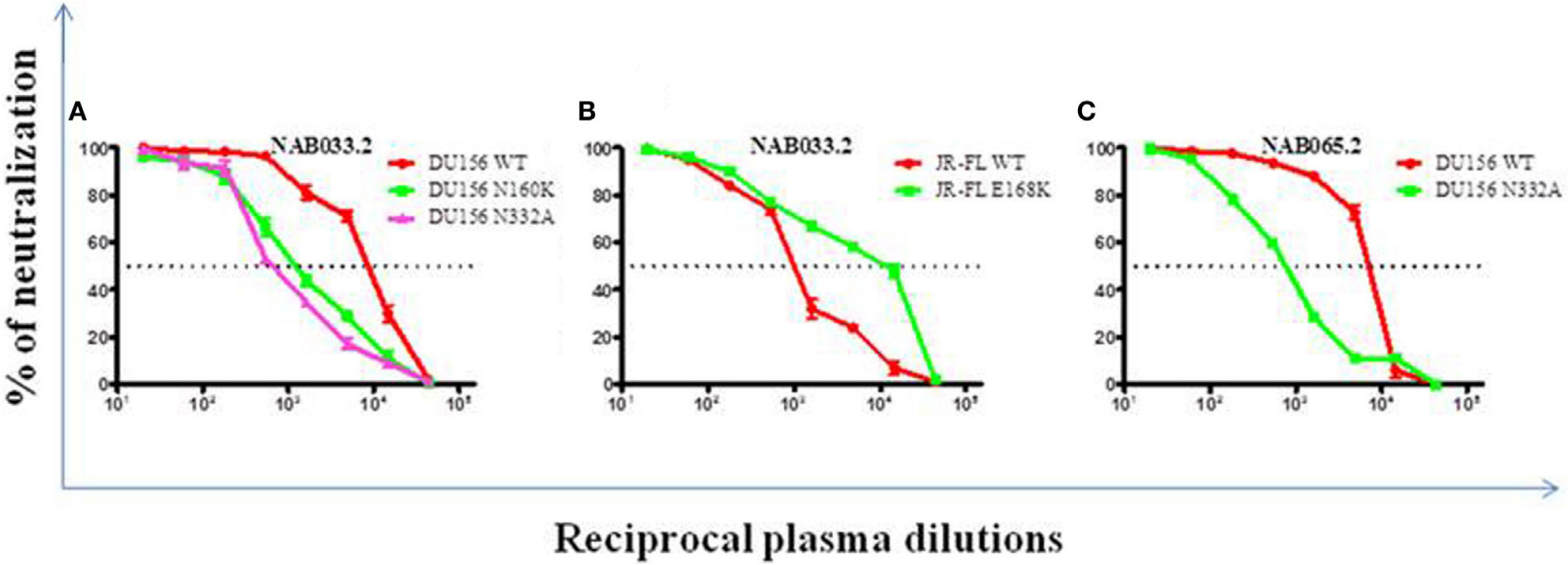
Neutralization analysis to identify glycan-dependent bNAbs. **(A)** Neutralization assay was performed against DU156WT, DU156N160K, and DU156N332A. **(B)** Presence of PG9/PG16 like bNAb specificity in sample NAB033 was confirmed by performing neutralization assay with JR-FL WT and JR-FL E168K. **(C)** Presence of PGT series like bNAbs in the sample NAB065 was confirmed by performing neutralization assay with DU156WT and DU156N332A. Each sample was tested in duplicate (1:20 to 1:43,740 dilutions), and the experiment was repeated on two independent occasions. ID_50_ values were calculated using non-linear regression with variable loops using Graph pad prism 5.0. software.

Previously, we had identified four BCN plasma samples with neutralization specificities similar to the PG9/PG16 class of bNAbs (1). However, second time point sample could be collected only from one of the four individuals. This sample (NAB033) was tested in a neutralization assay with pseudoviruses DU156WT and DU156 N160K. Strong neutralization of the DU156 WT pseudovirus was observed (Figure 6A; Table 5) with a 6.7-fold lesser neutralization of the DU156 N160K mutant as compared to the wild type, indicating the persistence of significant amount of antibodies that target the N160 glycan of the HIV-1 Env in this sample.

**Table 5:** Neutralization activity of BCN samples against DU156 pseudovirus with mutations in peptidoglycans targeted by the PG9/PG16 and PGT series of bNAbs.

Sample ID	ID_50_			Fold change^#^	
		
DU156WT	N160K	N332A	N160K	N332A
NAB033.1	623.8	580	310.9	**1.1**	**2.0**
NAB033.2	9601	1421	703.9	6.7*	13.6*
NAB065.1	468.2	609.4	184.4	0.8	**2.5**
NAB065.2	8165	ND	882.6	NA	9.2*
HHP	<20	<20	<20	NA	NA

^#^Fold change = ID_50_ of HIV-1 _DU156WT_/ID_50_ of HIV-1 _DU156 mutant_. Values with fold changes ≥3.0 are highlighted in bold format. ND indicates not done; NA indicates not applicable. *p < 0.001 when first time point results were compared to second time point results.

Neutralization assay was also performed with JR-FL WT and JR-FL E168K mutant viruses. The PG9/PG16 series of bNAbs are unable to neutralize the JR-FL WT HIV-1 strain because the glycan at position N160 is hidden by the glutamic acid (E) present at position 168 in this virus. The PG9/PG16 bNAbs show strong neutralization of the JR-FL pseudovirus when the glutamic acid at position 168 is replaced with lysine (K) in the JR-FL back bone (JR-FL E168K) (3). It was observed that the plasma of NAB033 showed >10-fold stronger neutralization of the JR-FL E168K mutant than the JR-FL WT pseudovirus, providing further evidence to support the presence of significant amounts of PG9/PG16-like antibodies in this sample (Figure 6B; Table 6). Interestingly, we observed significant increase in the neutralization property specifically targeting the N160 glycan of the HIV-1 Env at the second time point in NAB033 as compared to the first time point (*p* < 0.001) (Figure S2 in Supplementary Material).

**Table 6:** Neutralization activity against HIV-1 JR-FL WT and E168K mutant pseudoviruses.

Sample ID	ID_50_		Fold change^#^
		
	JR-FL WT	E168K	E168K
NAB033.1	1,008	4,999	**4.96**
NAB033.2	1,149	11,988	10.4*
HHP	<20	<20	NA

^#^Fold change calculated by ID_50_ of JR-FL E168K/ID_50_ of JR-FL WT. Neutralization sensitivity >3.0-fold are highlighted in bold format. *p < 0.001 when first time point results were compared to second time point results.

### Increase in PGT Series—Like Neutralization Specificity Over Time

In the initial screening, we identified seven BCN samples showing neutralization specificity similar to that of the PGT series of bNAbs (1). However, we could collect second time point sample only from two of these individuals (NAB033 and NAB065). The plasma samples were tested in a neutralization assay with the DU156 WT and DU156 N332A mutant pseudoviruses generally used for this purpose (Figures 6A,C). It was observed that both samples showed significantly stronger neutralization of the DU156 WT pseudovirus than the DU156 N332A mutant; the neutralization activity of NAB033 and NAB065 being 13.6- and 9.2-fold lower with the DU156 N332A mutant as compared to DU156 WT virus (Table 6). Interestingly, both samples showed a significant increase in neutralization property specifically targeting the N332 glycan of the HIV-1 Env in these two samples when compared to the initial time point (*p* < 0.001) (Figure S2 in Supplementary Material).

### Increase in Antibodies Specific to Native HIV-1 Env Trimers

**Figure 7:**
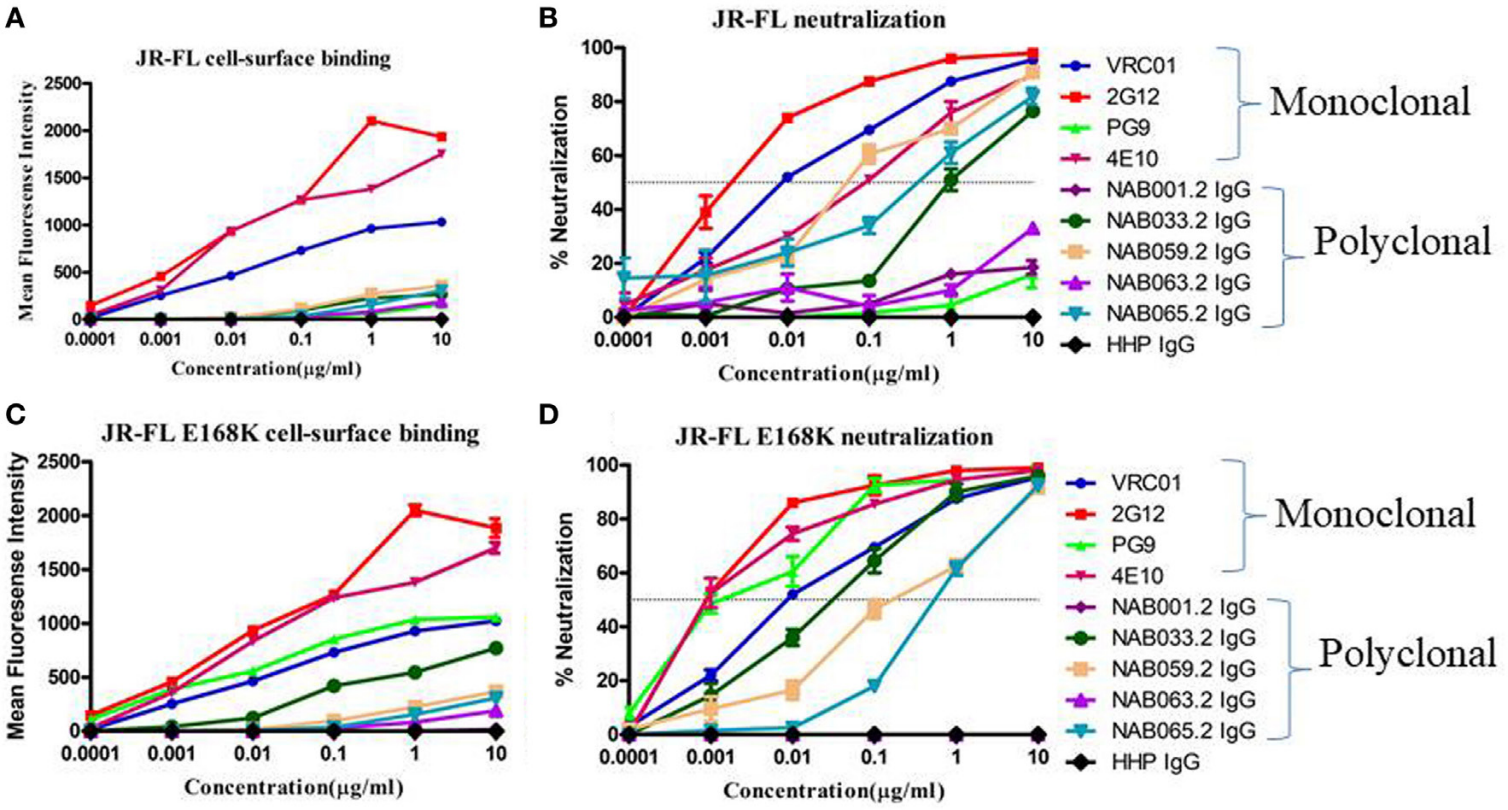
Cell-surface binding and neutralization analysis of IgG eluted from BCN samples. **(A,C)** Cell-surface binding assay with IgG eluted from BCN samples and control mAbs with JR-FL WT and JR-FL E168K mutant Env trimers expression on the surface of 293 T cells as determined by their mean fluorescence intensity in flow cytometry. **(B,D)** Neutralization activity of IgG from BCN samples and control mAbs against JR-FL WT and JR-FL E168K mutant pseudoviruses. VRC01, 2G12, PG9, and 4E10 were included for comparison as positive controls and HHP IgG was used as negative control. Each experiment was performed in duplicate on two independent occasions.

To further characterize the neutralization property of HIV-1 Env-specific antibodies present in the samples, we eluted Env trimer specific (93IN101-gp145 trimer) polyclonal IgG from the plasma and performed cell-surface binding as well as neutralization assays with JR-FL WT and JR-FL E168K mutant Env protein and pseudoviruses, respectively. Analysis of the results of the cell-surface binding assay with eluted IgG showed that one of the samples (NAB033) showed stronger binding to the JR-FL E168K mutant (30.49-fold) than to the JR-FL WT Env (Figure 7; Table 7), similar to what was observed with the positive control mAb PG9, which also exhibited significantly stronger binding (142.43-fold) to JR-FL mutant than to JR-FL WT. We then compared the neutralization potency of the eluted IgG with that of known mAbs using HIV-1 JR-FL WT and single mutant JR-FL E168K pseudoviruses. We observed that the median inhibitory concentration (IC_50_) of NAB033.2 with JR-FL E168K mutant was 12.4-fold lower (i.e., more potent neutralization) than the IC_50_ with JR-FL WT, as was also seen with the mAb PG9 (Figure 7; Table 7). Collectively our results suggest strongly that the sample NAB033 contains potent PG9/PG16 like bNAbs.

**Table 7:** Comparison of cell-surface binding (EC_50_) and neutralization activity (IC_50_) of IgG eluted from BCN samples against JR-FL WT and JR-FL E168K mutant.

Antibody	JR-FL WT		JR-FL E168K		JR-FL EC_50_/IC_50_	E168K EC_50_/IC_50_	Fold change*	
					
	Cell-surface binding	Neutralization	Cell-surface binding	Neutralization			JR-FL EC_50_/E168K EC_50_	JR-FL IC_50_/E168K IC_50_
						
	EC_50_ (μg/ml)	IC_50_ (μg/ml)	EC_50_ (μg/ml)	IC_50_ (μg/ml)				
VRC01	0.0152	0.069	0.01502	0.0702	0.22	0.21	1.01	0.98
2G12	0.02807	0.0072	0.02555	0.007294	3.90	3.50	1.10	0.99
PG9	1.34	>10	0.009408	0.006812	ND	1.38	**142.43**	ND
4E 10	0.01637	0.007	0.01605	0.008399	2.34	1.91	1.02	0.83
NAB001.2 IgG	3.155	>10	3.155	>10	ND	ND	1.00	ND
NAB033.2 IgG	3.393	1	0.1113	0.08038	3.39	1.40	**30.49**	**12.44**
NAB059.2 IgG	0.2528	0.91	0.4426	0.91	0.28	0.49	0.57	1.00
NAB063.2 IgG	1.12	>10	1.125	>10	ND	ND	1.00	ND
NAB065.2 IgG	0.91	1	0.91	0.95	0.91	0.96	1.00	1.05
HHP IgG	0.00	0.00	0.00	0.00	0.00	0.00	0.00	0.00

Cell surface binding and neutralization assays were performed with pIndie-gp145 trimer specific IgG eluted from BCN samples. VRC01, 2G12, PG9, and 4E10 monoclonal antibodies were included for the comparison of class specific bNAbs. HHP IgG was used as the negative control. This experiment was performed in duplicates and median inhibitory concentration (IC_50_) and effective concentration (EC_50_) values were calculated by non-linear regression method in graph pad prism. *Fold change = IC_50_ or EC_50_ of HIV-1 _JR-FL WT_/IC_50_ or EC_50_ of HIV-1 _JR-FL mutant_. Values >3.0 are highlighted in bold format. ND indicates not determined because IC_50_ values were >10 μg/ml.

## Discussion

We recently published a study in which we identified 12 HIV-1 subtype C infected individuals whose plasma showed broad and potent cross-clade neutralization (BPCN) property (1). Subsequently we obtained a follow-up sample from a subset of these individuals after a four-year interval, to evaluate the evolution of the host NAb response over time. Few studies suggest that evolution of viral variants as well as bNAbs takes place simultaneously, and that acquisition of neutralization breadth and potency continue during ongoing HIV-1 infection (48, 49). However, the evolution of high level of neutralization breadth and potency may take several years in order to attain special features not found in most Ab responses, i.e., neutralization of most of the globally circulating HIV-1 strains at very minimal concentrations (in other words, the Ab concentration needed to achieve 50% neutralization should be less than 1 µg/ml) (17). Liao et al. in 2013 had reported increase in neutralization potency in an African HIV-infected individual (CH505), in a longitudinal study that compared baseline and follow-up samples of those with broad clade neutralization ability and suggested a maturation of the broadly NAb response (48). More recently, Patil et al. (33) reported similar observations in a HIV-infected individual from India. In the present study, we made similar observations of increase in neutralization potency of BCN samples during follow-up as demonstrated by an increase in ID_50_ values. Interestingly, we observed that one sample (NAB059) which exhibited strong neutralization potency at the time of initial screening (1), showed a tremendous increase in neutralization potency during follow-up with GMT > 7100 (6.75-fold), prompting us to categorize this individual as an elite neutralizer. The remaining four samples (NAB001, NAB033, NAB063, and NAB065) also showed a significant increase in the neutralization potency of the follow-up samples with GMT ranging between 1,100 and 1,800 as compared to 100 and 400 at the initial time point.

We went on to characterize the neutralization specificity in the study subjects in a systematic manner. We first performed a PepScan analysis to determine if any of the samples had acquired newer antibody responses. We found that NAB059 and NAB033 had evolved additional antibody responses with binding reactivity spread across the C2, V4, C4, C5, HR2, and CT of the envelope. Interestingly the plasma (NAB059) retained binding reactivity to the Kennedy epitope (KE; PRGPDRPEGIEEEGGERDRDRS) present in the transmembrane region of gp41 (amino acids 731 to 752as on HXB2) (50, 51), as seen during the initial testing (1). Since most bNAbs are known to bind strongly to conformational epitopes on the HIV-1 envelope than to linear epitopes, and bring about virus neutralization (38, 52), we went on to analyze the antibody response with well-characterized conformational and native forms of the Env protein. We found a significant increase in the binding reactivity of one of the follow-up samples (NAB059) to 93IN101 gp145 trimer, dimer, HXB2 gp120 monomer, and NL4-3 gp41 monomer (*p* < 0.05), indicating the evolution of a more robust antibody response to the native forms of the envelope glycoprotein over time. Similarly the follow-up sample of NAB033 also showed a significant increase in binding reactivity to the NL4-3 gp41 monomer (*p* < 0.001).

Further, we characterized the samples for CD4BS neutralization specificity. Three of the five BCN samples (NAB033, NAB059, and NAB063) were found to possess CD4BS-specific antibody response at the initial time point. In the present analysis, the binding reactivity of two of the three samples (NAB033 and NAB059) was found to have increased significantly with the RSC3 protein (*p* < 0.001). RSC3 WT and RSC3Δ371I/P363N recombinant proteins have been employed widely for the identification of CD4BS NAbs from the HIV-1-infected individuals (1, 7, 53). It was of particular interest to note that these two subjects also maintained a good CD4 + T cell count > 350 cells/mm^3^ and continued to be naïve to ART, pointing to good correlation between positive evolution of the bNAb response to the CD4BS with clinical outcome. This makes these samples worthy of further investigation for isolation of potent bNAbs that can be used as therapeutic tools. Previous studies have reported that Indian HIV-1 subtype C strains show considerable molecular differences in their envelope as compared to the other HIV-1 subtypes around the globe (53), and that RSC3 reactive antibodies mediating broad neutralization alone do not include all CD4BS antibodies (54). This has necessitated the use of additional recombinant gp120 (rgp120) core molecules for identification of CD4BS antibodies (20). Saha et al. (43) and Bhattacharyya et al. (44) designed a protein called CD4OD, with alterations in the CD4OD of the HIV-1 subtype B Env protein, such that it also binds CD4BS antibodies present in HIV-1 subtype C infected individuals from India and other parts of the globe. When screened with the CD4OD recombinant protein, we observed that a third sample (NAB063), also exhibited stronger binding reactivity to the CD4OD protein as compared to the initial time point (Figure 5B). Bhattacharyya et al. (45) have reported that CD4BS neutralizing antibodies and not n-NAbs show specific binding reactivity to the CD4OD protein, indicating an increase in CD4BS specific antibody response over time in individuals having this class of bNAbs.

The Env trimer is known to be covered in a dense array of glycans constituting about half of its mass (2, 3, 10, 17). The glycans play a critical role in viral escape from the host immune response. Two major classes of bNAbs that target glycan-dependent epitopes have been identified; these include an epitope in the V1/V2 loop containing a glycan at N160 (PG9/PG16-like), and an epitope in the V3 loop containing a glycan at N332 (PGT 128-series). We had identified in our previous study 4 BCN plasma samples (NAB016, NAB033, NAB062 and NAB069) with PG9/PG16-like NAb specificity (1). However, only one of these 4 samples (NAB033) could be obtained for follow-up analysis. When this sample was tested for neutralization against HIV-1 subtype C DU156WT and DU156N160K pseudoviruses, it was observed that there was a fourfold increase in the neutralization efficiency of DU156 WT and 6.7-fold decrease in neutralization efficiency of DU156N160K mutant virus as compared to the first time sample. It was also observed that there was a >fivefold increased dependency on glycan at position 160 in the follow-up sample (6.7-fold) as compared to the first time sample (1.1-fold). Since the PG9/PG16 class of bNAbs are known to strongly neutralize the mutant JR-FL E168K strain but not JR-FL WT (3, 55), we also performed a neutralization assay with the JR-FL WT and JR-FL E168K mutant pseudoviruses to confirm the presence of PG9/PG16-like neutralization property, and found a >10-fold neutralization of the mutant JR-FL E168K than the JR-FL WT. Comparison of the obtained results with that of the first time point sample (1), showed a >onefold increase in neutralization of the JR-FL E168K mutant pseudovirus, indicating that this sample had evolved a stronger bNAb response to the epitope comprising N160 glycan of the HIV-1 Env.

Two of the seven BCN samples (NAB033 and NAB065), which exhibited PGT series-like neutralization property in our earlier study (1), were available for follow-up analysis. These samples were tested with DU156WT and DU156 N332A mutant pseudoviruses that are widely employed for the screening of this class of bNAbs. The two plasma samples neutralized the WT virus with 13.6- and 9.2-fold greater potency than the mutant DU156N332A pseudovirus. When compared to the results of the initial sample, the follow-up samples of NAB033 and NAB065 showed a >threefold and >onefold increase in bNAb specificity toward the N332 glycan, respectively.

Previous studies have reported a strong correlation between antibody responses to the cell-surface expressed native Env trimers and neutralization potency in other systems (56). In order to correlate the bNAbs binding response with neutralizing potency in the present study, we isolated polyclonal IgG from all the five BCN samples using pIndie gp145 trimeric protein coated My One Dynabeads. IgG was eluted and tested for neutralization potency and cell-surface binding affinity to HIV-1 JR-FL WT and its single mutant JR-FL E168K. We observed that median IC_50_ values were lower than EC_50_ values, indicating the highly potent nature of the neutralizing antibodies in these samples. Overall, our results suggest that NAB033 has strong neutralization property similar that of the PG9 class of bNAbs, prompting us to further characterize the antibodies in this sample.

To summarize, we observed a positive evolution of neutralization potency in most of the second time samples as seen by increase in ID_50_ values over time. Interestingly, the sample NAB059 that had the greatest increase in potency of neutralization also showed an increase in CD4BS specific reactivity during follow-up and this was associated with a stable CD4+ T cells count >350 cells/mm^3^, lower plasma viral load, and good clinical correlation making this a very interesting case to pursue for the isolation of potent bNAbs that can be used as potential therapeutic tools. Overall, our findings are quite encouraging as they suggest that the potency of the BCN response, once initiated, increases and matures with time.

## Ethics Statement

The study was approved by the Institutional Ethics Committee of the National Institute for Research in Tuberculosis (NIRT IEC No: 2011001) and all experiments were performed in accordance with relevant guidelines and regulations. Sample collection was done after obtaining written informed consent from the study participants.

## Author Contributions

NC, SS, ST, RV, KS, RD, and LH participated in the study design. NC, MS, PS, KM, and NK collected and processed samples, NC, HB, and BA performed the experiments and data analysis. NC and LH drafted the paper. All authors have read and approved the final manuscript.

## Conflict of Interest Statement

The authors declare that the research was conducted in the absence of any commercial or financial relationships that could be construed as a potential conflict of interest.
