# Supplementary material for: Evolution of Neutralization Response in HIV-1 Subtype C-Infected Individuals Exhibiting Broad Cross-Clade Neutralization of HIV-1 Strains
Source: Front Immunol. 2018 Mar 27;9:618. doi: 10.3389/fimmu.2018.00618 (PMC5890096; doi:10.3389/fimmu.2018.00618)
Supplement: Supplementary file 1 [file data_sheet_1.DOCX]

**Supplementary Materials for**

**Evolution of Neutralization response in HIV-1 subtype C Infected Individuals exhibiting Broad Cross-clade Neutralization of HIV-1 strains**

Narayanaiah Cheedarla^1^, Babu Hemalatha^1^, Brahmaiah Anangi^2^, Kannan Muthuramalingam^1^, Murugesan Selvachithiram^1^, Sathyamurthi Pattabiraman^1^, Nandagopal Kailasam^3^, Raghavan Varadarajan^4^, Soumya Swaminathan^1^, Srikanth Prasad Tripathy^1^, Kalyanaraman Vaniambadi S^5^, Ramanathan Vadakkupattu D^1^, Luke Elizabeth Hanna^1*^

^1^Department of HIV/AIDS, National Institute for Research in Tuberculosis, Chennai, India – 600031, ^2^Molecular Virology Laboratory, Molecular Biology and Genetics Unit, Jawaharlal Nehru Centre for Advanced Scientific Research, Bangalore, India, ^3^ART Center, Kilpauk Medical College and Hospital, Chennai, 600010, India, ^4^Molecular Biophysics Unit, Indian Institute of Science, Bangalore 560012, India, ^5^Advanced Bioscience Laboratories Inc., Rockville, MD, 20850, USA.

**Key points:** HIV-1, broadly Neutralizing antibodies, Indian HIV-1 subtype C infection, evolution of neutralization response, CD4 binding site, glycan-dependent neutralization.

* Correspondence and requests for materials should be addressed to

Dr. Luke Elizabeth Hanna, (E-mail: [hanna@nirt.res.in](mailto:hanna@nirt.res.in) Tel. +91-44-2836-9597)


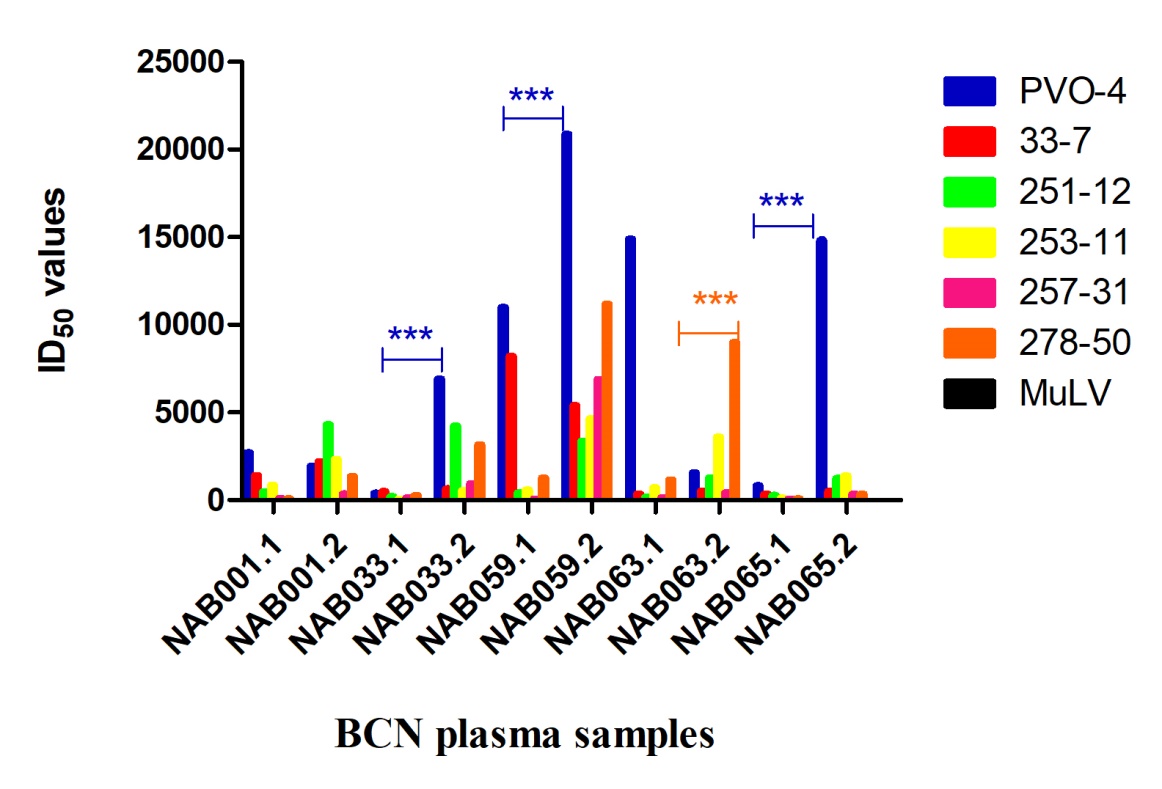


Supporting figure S1: Comparison of neutralization potency against tier-3 pseudoviruses. ID50 values were used for comparison of neutralization potency [36]. This experiment was performed in duplicate on two independent occasions and average values were taken for the comparison.


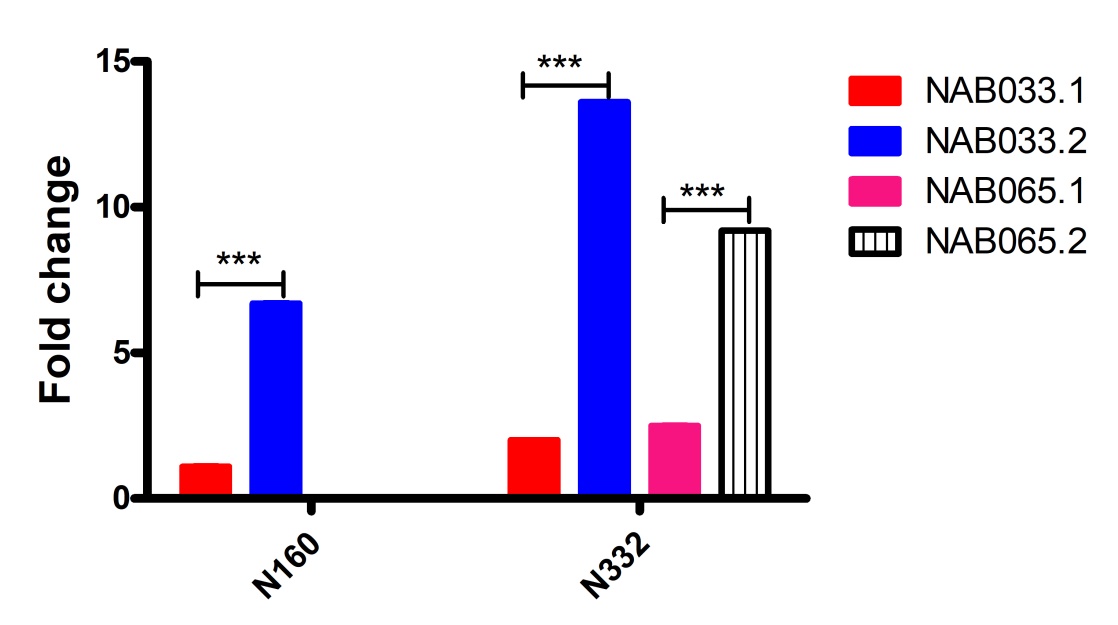


Supporting figure S2: Comparison of neutralization activity against glycans at position N160 and N332 on HIV-1 envelope glycoprotein. Fold change in the neutralization calculated for the initial time point [36] and follow-up samples. This experiment was performed in duplicate on two independent occasions and average values were taken for the comparison.

**Supplementary Table S1: Pseudoviruses used in this study**

| **S.No** | **Name of the pseudovirus** | **Subtype** | **Class of tier** |
| --- | --- | --- | --- |
| 1 | 33-7 | A/G | 3 |
| 2 | 251.18 | A/G | 3 |
| 3 | 253.11 | A/G | 3 |
| 4 | 278.50 | A/G | 3 |
| 5 | PVO.4 | B | 3 |
| 6 | 257 | A/G | 3 |
| 7 | DU156 | C | 2 |
| 8 | DU156 N160K | C | 2 |
| 9 | DU156 N332A | C | 2 |
| 10 | JR-FL | B | 2 |
| 11 | JR-FL E168K | B | 2 |
| 12 | MuLV | - | - |

Supplementary table S2: List of peptides used in the PepScan ELISA

| **S.NO** | **Linear Overlapping peptides of 93IN101 gp160 protein** |
| --- | --- |
| 1 | GGNLWVTVYYGVPVW |
| 2 | WVTVYYGVPVWKEAK |
| 3 | YYGVPVWKEAKTTLL |
| 4 | PVWKEAKTTLLCAST |
| 5 | EAKTTLLCASDAKAY |
| 6 | TLLCASDAKAYERAV |
| 7 | ASDAKAYEREBHNVW |
| 8 | KAYEREVHNVWATHA |
| 9 | REVHNVWATHACVPT |
| 10 | NVWATHACVPTDPNP |
| 11 | THACVPTDPNPQEIV |
| 12 | VPTDPNPQEIVLGNV |
| 13 | PNPQEIVLGNVTENF |
| 14 | EIVLGNVTENFNMWK |
| 15 | GNVTENFNMWKNDMB |
| 16 | ENFNMWKNDNMVDQMH |
| 17 | MWKNDMVDQMHEDVI |
| 18 | DMVDQNHEDVISLWD |
| 19 | QMHEDVISLWDQSLK |
| 20 | DVISLWQSLKPCVK |
| 21 | LWDQSLKPCVKLTPL |
| 22 | SLKPCVKLTPLCVTL |
| 23 | CVKLTPLCVTLECRN |
| 24 | TPLCVTLECRNVSRN |
| 25 | VTLECRNVSRNVSSY |
| 26 | CRNVSRNVSSYNTYN |
| 27 | SRNVSSYNTYNGSVE |
| 28 | SSYNTYNGSVEEIKN |
| 29 | TYNGSVEEIKNCSFN |
| 30 | SVEEIKNCSFNATPE |
| 31 | IKNCSFNATPEVRDR |
| 32 | SFNATPEVRDRKQRM |
| 33 | TPEVRDRKQRMYALF |
| 34 | RDRKQRMYALFYGLD |
| 35 | QRMYALFYGLDIVPL |
| 36 | ALFYGLDIVPLNKKN |
| 37 | GLDIVPLNKKNSSEN |
| 38 | VPLNKKNSSENSSEY |
| 39 | KKNSSENSSEYRLIN |
| 40 | SENSSEYRLINCNTS |
| 41 | SEYRLINCNTSAITQ |
| 42 | LINCNTSAITQACPK |
| 43 | NTSAITQACPKVTFD |
| 44 | ITQACPKVTFDPIPI |
| 45 | CPKVTFDPIPIHYCA |
| 46 | TFDPIPIHYCAPAGY |
| 47 | IPIHYCAPAGYAILK |
| 48 | YCAPAGYAILKCNNK |
| 49 | AGYAILKCNNKTFNG |
| 50 | ILKCNNKTFNGTGPC |
| 51 | NNKTFNGTGPCNNVS |
| 52 | FNGTGPCNNVSTVQC |
| 53 | GPCNNVSTVQCTHGI |
| 54 | NVSTVQCTHGIKPVV |
| 55 | VQCTHGIKPVVSTQL |
| 56 | HGIKPVVSTQLLLNG |
| 57 | PVVSTQLLLNGSLAE |
| 58 | TQLLLNGSLAEGEII |
| 59 | LNGSLAEGEIIIRSE |
| 60 | LAEGEIIIRSENLTN |
| 61 | EIIIRSENLTNNVKT |
| 62 | RSENLTNNVKTIIVH |
| 63 | LTNNVKTIIVHLNQS |
| 64 | VKTIIVHLNQSVEIV |
| 65 | IVHLNQSVEIVCTRP |
| 66 | NQSVEIVCTRPNNNT |
| 67 | EIVCTRPNNNTRKSI |
| 68 | TRPNNNTRKSIRIGP |
| 69 | NNTRKSIRIGPGQTF |
| 70 | KSIRIGPGQTFYATG |
| 71 | IGPGQTFYATGDIIG |
| 72 | QTFYATGDIIGDIRQ |
| 73 | ATGDIIGDIRQAHCN |
| 74 | IIGDIRQAHCNISRD |
| 75 | IRQAHCNISRDKWNE |
| 76 | HCNISRDKWNETLQR |
| 77 | SRDKWNETLQRVGKK |
| 78 | WNETLQRVGKKLAEH |
| 79 | LQRVGKKLAEHFHNK |
| 80 | GKKLAEHFHNKTIKF |
| 81 | AEHFHNKTIKFASSS |
| 82 | HNKTIKFASSSGGDL |
| 83 | IKFASSSGGDLEITT |
| 84 | SSSGGDLEITTHSFN |
| 85 | GDLEITTHSFNCRGE |
| 86 | ITTHSFNCRGEFFYC |
| 87 | SFNCRGEFFYCNTSG |
| 88 | RGEFFYCNTSGLFNG |
| 89 | FYCNTSGLFNGTYMP |
| 90 | TSGLFNGTYMPTYMP |
| 91 | FNGTYMPTYMPNGTE |
| 92 | YMPTYMPNGTESNSN |
| 93 | YMPNGTESNSNSTIT |
| 94 | GTESNSNSTITIPCR |
| 95 | NSNSTITIPCRIKQI |
| 96 | TITIPCRIKQIINMW |
| 97 | PCRIKQIINMWQEVG |
| 98 | KQIINMWQEVGRAMY |
| 99 | NMWQEVGRAMYAPPI |
| 100 | EVGRAMYAPPIAGNI |
| 101 | AMYAPPIAGNITCTS |
| 102 | PPIAGNITCTSNITG |
| 103 | GNITCTSNITGLLLV |
| 104 | CTSNITGLLLVHDGG |
| 105 | ITGLLLVHDGGIKEN |
| 106 | LLVHDGGIKENDTEN |
| 107 | DGGIKENDTENKTEI |
| 108 | KENDTENKTEIFRPG |
| 109 | TENKTEIFRPGGGDM |
| 110 | TEIFRPGGGDMRDNW |
| 111 | RPGGGDMRDNWRSEL |
| 112 | GDMRDNWRSELYKYK |
| 113 | DNWRSELYKYKVVEI |
| 114 | SELYKYKVVEIKPLG |
| 115 | KYKVVEIKPLGVAPT |
| 116 | VEIKPLGVAPTAAKR |
| 117 | PLGVAPTAAKRRVVE |
| 118 | APTAAKRRVVEREKR |
| 119 | AKRRVVEREKRAVGI |
| 120 | VVEREKRAVGIGAVF |
| 121 | EKRAVGIGAVFLGFL |
| 122 | VGIGAVFLGFLGAAG |
| 123 | AVFLGFLGAAGSTMG |
| 124 | GFLGAAGSTMGAASI |
| 125 | AAGSTMGAASITLTA |
| 126 | TMGAASITLTAQARQ |
| 127 | ASITLTAQARQLLSG |
| 128 | LTAQARQLLSGIVQQ |
| 129 | ARQLLSGIVQQQSNL |
| 130 | LSGIVQQQSNLLRAI |
| 131 | VQQQSNLLRAIEAQQ |
| 132 | SNLLRAIEAQQHLLQ |
| 133 | RAIEAQQHLLQLTVW |
| 134 | AQQHLLQLTVWGIKQ |
| 135 | LLQLTVWGIKQLQTR |
| 136 | TVWGIKQLQTRVLAI |
| 137 | IKQLQTRVLAIERYL |
| 138 | QTRVLAIERYLKDQQ |
| 139 | LAIERYLKDQQLLGI |
| 140 | RYLKDQQLLGIWGCS |
| 141 | DQQLLGIWGCSGKLI |
| 142 | LGIWGCSGKLICTTA |
| 143 | GCSGKLICTTAVPWN |
| 144 | KLICTTAVPWNSSWS |
| 145 | TTAVPWNSSWSNKTQ |
| 146 | PWNSSWSNKTQSEIW |
| 147 | SWSNKTQSEIWNNMT |
| 148 | KTQSEIWNNMTWMQW |
| 149 | EIWNNMTWMQWDREV |
| 150 | NMTWMQWDREVSNYT |
| 151 | MQWDREVSNYTNIIY |
| 152 | REVSNYTNIIYSLLE |
| 153 | NYTNIIYSLLEESQN |
| 154 | IIYSLLEESQNQQEK |
| 155 | LLEESQNQQEKNEKD |
| 156 | SQNQQEKNEKDLLAL |
| 157 | QEKNEKDLLALDSWK |
| 158 | EKDLLALDSWKNLWS |
| 159 | LALDSWKNLWSWFDI |
| 160 | SWKNLWSWFDITNWL |
| 161 | LWSWFDITNWLWYIK |
| 162 | FDITNWLWYIKIFIM |
| 163 | NWLWYIKIFIMIVGG |
| 164 | YIKIFIMIVGGLIGL |
| 165 | FIMIVGGLIGLRIIF |
| 166 | VGGLIGLRIIFAVLS |
| 167 | IGLRIIFAVLSIVNR |
| 168 | IIFAVLSIVNRVRQG |
| 169 | VLSIVNRVRQGYSPL |
| 170 | VNRVRQGYSPLSFQT |
| 171 | RQGYSPLSFQTLTPN |
| 172 | RQGYSPLSFQTPRGP |
| 173 | SPLSFQTPRGPDRLG |
| 174 | FQTPRGPDRLGRIEE |
| 175 | RGPDRLGRIEEEGGE |
| 176 | RLGRIEEEGGEQDKD |
| 177 | IEEEGGEQDKDRSIR |
| 178 | GGEQDKDRSIRLVNG |
| 179 | DKDRSIRLVNGFLAL |
| 180 | SIRLVNGFLALAWDD |
| 181 | VNGFLALAWDDLRNL |
| 182 | LALAWDDLRNLCLFS |
| 183 | WDDLRNLCLFSYHRL |
| 184 | RNLCLFSYHRLRDFI |
| 185 | LFSYHRLRDFISVAA |
| 186 | HRLRDFISVAARVVE |
| 187 | DFISVAARVVELLGR |
| 188 | VAARVVELLGRSSWE |
| 189 | VVELLGRSSWEALKY |
| 190 | LGRSSWEALKYLGSL |
| 191 | SWEALKYLGSLVQYW |
| 192 | LKYLGSLVQYWGLEL |

**Supplementary table S3: Binding reactivity of second time point plasma samples to linear peptides spanning the entire 93IN101 gp160 protein**

| **Regions of gp160** | **NAB001.2** | **NAB033.2** | **NAB059.2** | **NAB063.2** | **NAB065.2** | **Control (HHP)** |
| --- | --- | --- | --- | --- | --- | --- |
| C1 | 0.0 | 0.3 | 0.2 | 0.0 | 0.0 | 0.0 |
| V1 | 0.0 | 0.273 | 0.2 | 0.0 | 0.0 | 0.0 |
| V2 | 0.0 | 0.0 | 0.2 | 0.0 | 0.0 | 0.0 |
| C2 | 0.15 | 0.553 | 1.25 | 0.0 | 0.0 | 0.0 |
| V3 | 0.213 | 0.7 | 1.5 | 0.85 | 0.788 | 0.0 |
| C3 | 0.0 | 0.129 | 0.2 | 0.0 | 0.119 | 0.0 |
| V4 | 0.134 | 0.35 | 0.25 | 0.18 | 0.176 | 0.0 |
| C4 | 0.15 | 0.563 | 0.2 | 0.0 | 0.119 | 0.0 |
| V5 | 0.0 | 0.0 | 0.0 | 0.15 | 0.0 | 0.0 |
| C5 | 0.145 | 0.62 | 0.7 | 0.183 | 0.0 | 0.0 |
| HR1 | 0.0 | 0.0 | 0.16 | 0.0 | 0.0 | 0.0 |
| ID | 1.0 | 1.733 | 1.81 | 1.915 | 0.558 | 0.0 |
| HR2 | 0.0 | 0.474 | 0.2 | 0.0 | 0.0 | 0.0 |
| MPER | 0.128 | 0.0 | 0.16 | 0.0 | 0.0 | 0.0 |
| TM | 0.115 | 0.0 | 0.143 | 0.0 | 0.0 | 0.0 |
| KENNEDY | 0.0 | 0.15 | 2.12 | 0.0 | 0.162 | 0.0 |
| CT | 0.116 | 1.376 | 0.12 | 0.0 | 0.0 | 0.0 |

ELISA performed with a total of 192 peptides, 15 amino acid long with 11 amino acid overlapping peptides covering the entire 93IN101 envelope (gp160) protein. Plasma samples (n = 5) from BCN samples were tested at a dilution of 1:50 for the identification of binding reactivity. Healthy Human Plasma (HHP) was used as the negative control. This experiment was performed in duplicate on two independent occasions. Mean absorbance of HHP was 0.054 and two fold of HHP absorbance (0.162) was considered as positive reactivity. Normalized absorbance values are given in the table.
